# Rhythmical Photic Stimulation at Alpha Frequencies Produces Antidepressant-Like Effects in a Mouse Model of Depression

**DOI:** 10.1371/journal.pone.0145374

**Published:** 2016-01-04

**Authors:** Shinheun Kim, Sangwoo Kim, Arshi Khalid, Yong Jeong, Bumseok Jeong, Soon-Tae Lee, Keun-Hwa Jung, Kon Chu, Sang Kun Lee, Daejong Jeon

**Affiliations:** 1 Department of Bio and Brain Engineering, Korea Advanced Institute of Science and Technology, Yuseong, Daejeon, Republic of Korea; 2 Laboratory for Neurotherapeutics, Department of Neurology, Comprehensive Epilepsy Center, Biomedical Research Institute, Seoul National University Hospital (SNUH), Jongno-gu, Seoul, Republic of Korea; 3 Graduate School of Medical Science and Engineering, Korea Advanced Institute of Science and Technology, Yuseong, Daejeon, Republic of Korea; Karolinska Institute, SWEDEN

## Abstract

Current therapies for depression consist primarily of pharmacological agents, including antidepressants, and/or psychiatric counseling, such as psychotherapy. However, light therapy has recently begun to be considered as an effective tool for the treatment of the neuropsychiatric behaviors and symptoms of a variety of brain disorders or diseases, including depression. One methodology employed in light therapy involves flickering photic stimulation within a specific frequency range. The present study investigated whether flickering and flashing photic stimulation with light emitting diodes (LEDs) could improve depression-like behaviors in a corticosterone (CORT)-induced mouse model of depression. Additionally, the effects of the flickering and flashing lights on depressive behavior were compared with those of fluoxetine. Rhythmical flickering photic stimulation at alpha frequencies from 9–11 Hz clearly improved performance on behavioral tasks assessing anxiety, locomotor activity, social interaction, and despair. In contrast, fluoxetine treatment did not strongly improve behavioral performance during the same period compared with flickering photic stimulation. The present findings demonstrated that LED-derived flickering photic stimulation more rapidly improved behavioral outcomes in a CORT-induced mouse model of depression compared with fluoxetine. Thus, the present study suggests that rhythmical photic stimulation at alpha frequencies may aid in the improvement of the quality of life of patients with depression.

## Introduction

Depression, a common psychiatric disorder that affects approximately 121 million people worldwide, is considered one of the leading causes of disability [1]. Depression is associated with an increased prevalence of physical illnesses, decreased social functioning, and a high mortality rate which, in turn, result in significant social and economic burdens [2–4]. Epidemiological studies have shown that depression is common throughout the lifespan, as 20% of the world’s population has experienced a depressive episode at least once during their lifetime, and 2–5% of the world’s population has been affected by severe depression [5]. Current therapies for depression consist primarily of pharmacological agents, including antidepressants, and/or psychiatric counseling, such as psychotherapy. However, the outcomes associated with these therapies have not been always successful for patients with depression [5,6]; thus, additional or adjuvant therapeutic approaches are needed.

Light therapy has recently received an increasing amount of attention as a tool for the treatment of neuropsychiatric behaviors or depressive symptoms [7–13] because light and visual input have been shown to modulate mood and various cognitive behaviors [13–16]. Although light deprivation or irregular or aberrant light exposure, which causes abnormal light/dark cycles, can adversely affect mood and cognitive functioning [15–19], appropriate photic stimulation can potentially exert antidepressant effects in humans and in animal models of depression [12,20–25]. These findings suggest that the quality of photic stimulation is a crucial factor in the modulation of mood and cognition. A majority of studies evaluating light therapy have employed constant and prolonged bright light, which results in a long photoperiod of photic stimulation (i.e., constant bright-light therapy). Recently, rhythmical photic stimulation using flickering and/or flashing lights within specific frequency ranges has been shown to induce changes in the psychological state of patients as well as to produce beneficial effects on cognition and behavior [26,27]. Flickering photic stimulation at alpha frequencies from 8 to 13 Hz may alleviate pain or stress and may also improve behavioral performance [28–32]. However, the effects of flickering photic stimulation on depressive behavior have yet to be clarified, and the relationship between photic stimulation and the actions of antidepressant drugs on patients with depression has not been well documented.

Although it is difficult to mimic the exact nature of human depression in other animals, various rodent models that exhibit depression-like behaviors that can further the understanding of the pathophysiological mechanisms underlying human depression have been developed [33]. For example, mice receiving chronic exogenous exposure to corticosterone (CORT) via their drinking water exhibit stable stress-induced increases in the secretion of glucocorticoids, which mimic those of humans and also result in depression-like behaviors and neurochemical changes [34–36]. It has also been shown that chronic treatment with antidepressants, such as fluoxetine, can reverse the depression-like phenotype of this CORT model [35,37], and that aberrant functional brain connectivity is present in the same model [38]. Thus, the present study investigated the effects of flickering and flashing lights within a specific range of alpha frequencies on behaviors related to anxiety, locomotor activity, social interaction, and despair in a CORT-induced mouse model of depression. Furthermore, effect of flickering photic stimulation were compared with that of fluoxetine, an antidepressant drug from the class of selective serotonin reuptake inhibitor (SSRI) drugs.

## Materials and Methods

### Animals

**Fig 1:**
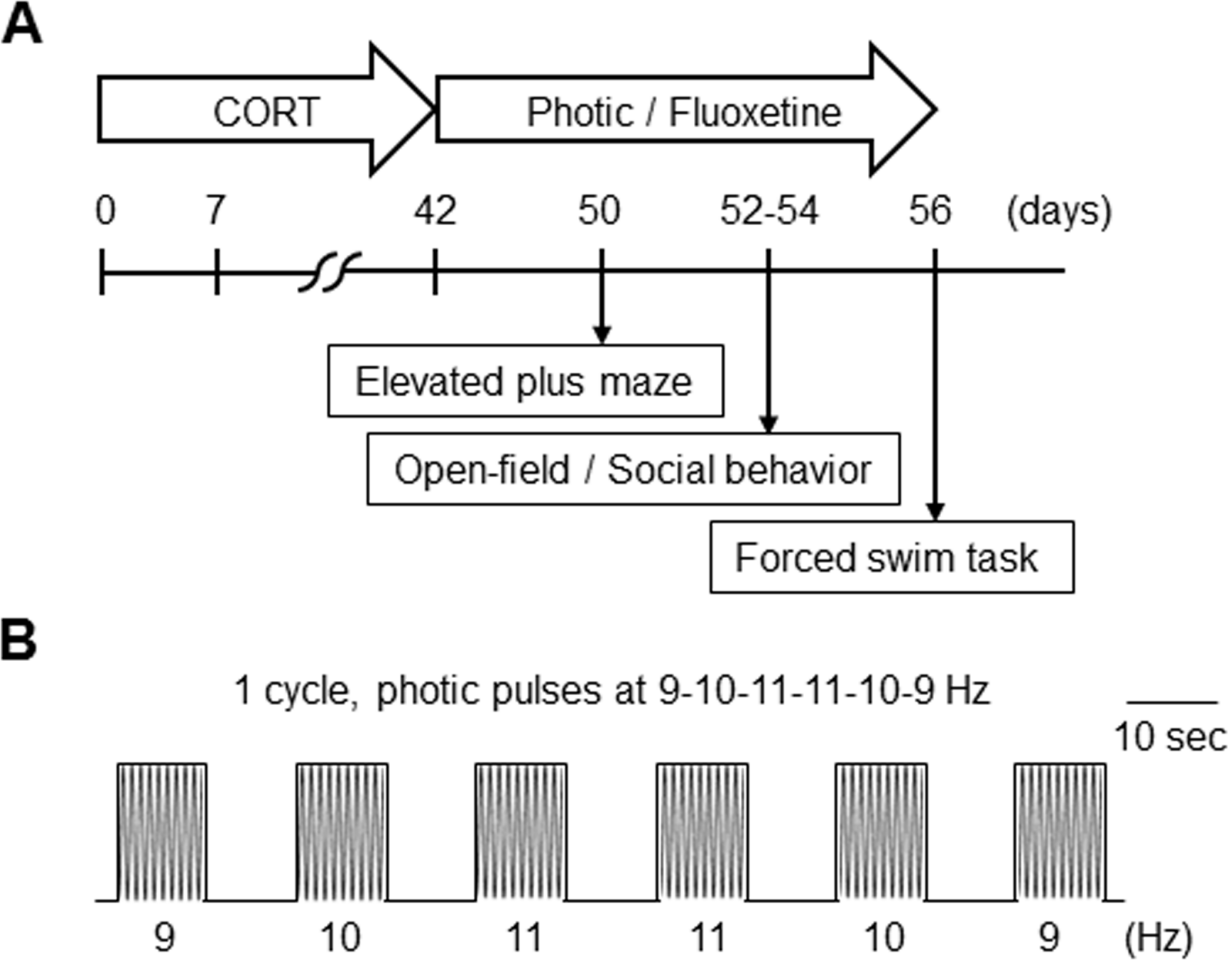
Experimental design. (A) Time schedule of experiments including behavioral testing and photic stimulation. Zero day indicates the onset of CORT administration. (B) Schematic illustration of rhythmic photic stimulation within the alpha frequency range. The flickering lights were delivered by LEDs, and a single cycle of photic stimulation contained six light pulses presented as follows: 9, 10, 11, 11, 10, and 9 Hz. This presentation was repeated 15 times per day for 8–14 days.

Male C57BL/6 mice (7–8 weeks old) were used. All subjects were housed in groups under a 12-h light/dark cycle (lights on at 08:00) and had ad libitum access to food and water. Animal studies were approved by the Institutional Animal Care and Use Committee at Korea Advanced Institute of Science and Technology University, and Biomedical Research Institute in Seoul National University Hospital (Protocol Number: 14–0210 and 14–0253). All efforts were made to minimize suffering. Carbon dioxide was used for euthanasia of mice. The experimental procedure is illustrated in Fig 1A.

### Generation of the CORT-induced mouse model of depression and drug treatments

The animal model of depression employed in the present study was generated using chronic exposure to CORT (Sigma; St. Louis, MO, USA), as previously described [35,38]. Briefly, mice received 35 μg/mL of CORT (equivalent to 5 mg/kg/day) dissolved in their drinking water along with 0.45% β-cyclodextrin (β-CD; Sigma) delivered in light-protected bottles that were replaced every 3 days for up to 42 days. After 42 days of exposure to CORT, the mice were randomly divided into four experimental groups: 1) mice were exposed to photic stimulation (photic-CORT group), 2) mice were exposed to fluoxetine (fluoxetine-CORT group), 3) mice were exposed to both the photic stimulation and fluoxetine (co-treatment group), and 4) mice were not treated with photic stimulation and fluoxetine (only-CORT group). The control group received only β-CD in their drinking water.

Fluoxetine (18 mg/kg/day; Anawa Trading; Wangen, Zurich, Switzerland) was administered to them in their drinking water. The fluoxetine was also delivered in light-protected opaque bottles but was replaced every 4 days until the end of experiment. The dose and duration were chosen based on the procedures of previous studies [35,39,40].

### Photic stimulation

Following the 42-day CORT exposure procedure, the mice received photic stimulation between 17:00 and 20:00 in a black-colored light-proof chamber (30 × 30 × 50 cm). The home cages of the mice without a cage lid were placed in the chamber, and the flickering and flashing light was provided by white light-emitting diode (LED) lamps (FLC-LTM 64 photic stimulator, Grass Technologies) located 28 ± 1 cm above the home cages. The mice were stimulated with light flashes (100 lux on the floor of the cage) for 5 ms at frequencies between 9 and 11 Hz. The rhythmic photic stimulation consisted of three alpha frequencies from 9 to 11 Hz that were alternatively applied in increments of 1 Hz; this was followed by 1-Hz decrements from 11 to 9 Hz (i.e., 9-10-11-11-10-9 Hz). The photic stimulation at each frequency was applied for 10 seconds and then followed by a 10-second period in which there was no photic stimulation. This cycle was repeated 15 times for a total trial period of 30 minutes per day. The photic stimulation procedure lasted for 8–14 days. The onset time and duration of photic stimulation were chosen according to the previous reports of light therapy [7–13,21,41–43]. The experimental protocol of photic stimulation is illustrated in Fig 1B.

### Behavioral tasks

Following the photic stimulation and fluoxetine treatment procedure, the mice performed behavioral tasks that assessed anxiety, locomotor activity, social behavior, and despair. All behavioral tests were video-recorded and conducted between 13:00 and 17:00 under a light intensity of 80 lux. There were no experimenters in the room during the behavioral tasks: the elevated plus maze, social interaction with a juvenile mouse, the open-field test, and the forced swim test. These were conducted 8 days, 10–12 days, 10–12 days, and 8 or 14 days after the photic stimulation procedure, respectively.

#### Elevated plus maze

For assessment of anxiety, elevated plus maze was performed as described previously [38,44,45]. This task was conducted after 8 days of photic stimulation or fluoxetine treatment. The maze was made of plastic and consisted of two white open arms (25 × 8 cm), two black enclosed arms (25 × 8 × 20 cm), and a central platform (8 × 8 × 8 cm) in the form of a cross. The maze was placed 50 cm above the floor. Mice were individually placed in the center with their heads directed toward one of the closed arms. The total time spent in each arm or in the center and the total number of entries into each arm was analyzed by video monitoring for 5 min. Only when all four paws crossed from the center into an arm, it was counted as an arm entry and used for measuring the amount of time spent in each arm.

#### Open-field task

To assess locomotor activity, open-field task was performed as described previously [38,44,45]. This task was conducted after 10–12 days of photic stimulation or fluoxetine treatment. The open-field box was made of white plastic (40 × 40 × 40 cm) and the open field was divided into a central field (center, 20 cm × 20 cm) and an outer field (periphery). Individual mice were placed in the periphery of the field and the paths of the animals were recorded with a video camera. The total distance traveled for 10 min and the time spent in the central area for first-5 min period were analyzed using a program (EthoVision XT, Noldus).

#### Social interaction with a juvenile mouse

To assess social interaction, a juvenile mouse was used. Male juvenile mice were used instead of adults to exclude any effect of mutual aggression [46]. This experiment was performed as described previously [45,47] and was conducted after 10–12 days of photic stimulation or fluoxetine treatment. A single subject mouse was allowed to roam freely in a new cage for 10 min (habituation). The cages used were identical to those in which the mice were normally housed. A novel juvenile (3–4 weeks old) male mouse was introduced to the cage and then allowed to roam freely for 5 min (test session). The following types of behavior were scored as social interaction: nose-to-nose sniffing, direct contact (pushing the snout or head underneath and crawling over or under the juvenile’s body), and following closely (within <1 cm) [48]. The total time spent engaging in social interaction behavior was quantified.

#### Forced swim task (FST)

FST for despair behavior was performed as described previously [35,38,49,50]. This task was conducted after 8 or 14 days of photic stimulation or fluoxetine treatment. Mice were placed individually in 2000 ml glass beakers filled with nearly 1400 ml of water (10 cm from the ground, with water temperature of 25 ± 1°C) and were allowed to swim freely for 6 min. The duration of immobility was measured during the last 4 min of the task. Duration of immobility is defined as immobile, floating state or minimal movement required for floating (for example, small, slow kicking of one paw only) and the absence of active swimming behavior.

### Statistical analysis

ANOVA was used to conduct multiple comparisons of means, followed by the Scheffe’s *post hoc* test. SPSS 21.0 (SPSS, Chicago, IL) was used for the statistical analyses. A p-value < 0.05 was considered to indicate statistical significance. All data are shown as means ± standard error of the mean (SEM).

## Results

### Mice receiving photic stimulation showed normal anxiety levels in the elevated plus maze

**Fig 2:**
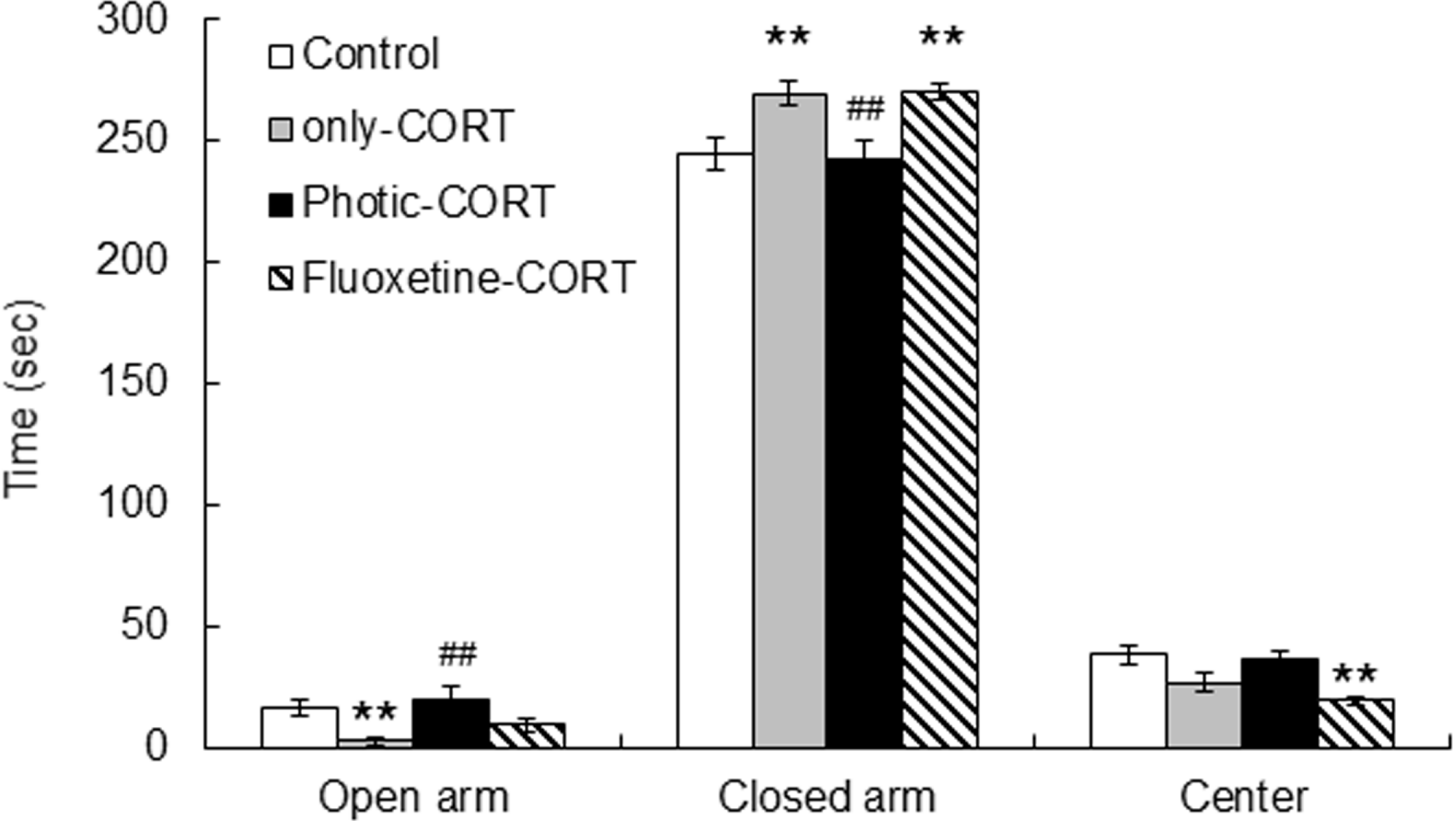
Effects of 8 days of treatment with photic stimulation or fluoxetine on the elevated plus maze task in the CORT-induced mouse model of depression. The only-CORT and fluoxetine-CORT groups spent more and less time in the closed and open arms, respectively, than the control group. However, the photic-CORT group spent a similar amount of time in the open and closed arms as the control group, which indicates that the photic-CORT group had a normal level of anxiety. *comparison with control group, **p < 0.01; ^#^comparison with only-CORT group, ^##^*p* < 0.01, Scheffe’s *post hoc* test.

The elevated plus maze task was conducted 8 days after photic stimulation or fluoxetine treatment to assess anxiety (Fig 2). There was a significant difference in the amount of time in the open (F_3, 64_ = 4.67, p < 0.01, One-way ANOVA) or closed arms (F_3, 64_ = 5.98, p < 0.01, One-way ANOVA) among the control, only-CORT group, photic-CORT, and fluoxetine-CORT groups. The only-CORT group (n = 19) spent less time (3.01 ± 1.45 sec) in the open arms than the control group (n = 20, 16.88 ± 3.58 sec) (p < 0.01, Scheffe’s *post hoc* test). In addition, the only-CORT group spent more time (269.80 ± 4.95 sec) in the closed arms than the control group (244.61 ± 6.34 sec) (p < 0.01, Scheffe’s *post hoc* test). These results indicate that the only-CORT group had increased levels of anxiety.

In contrast, the photic-CORT group (n = 18) spent a similar amount of time in the open (20.23 ± 5.24 sec) and closed (242.73 ± 7.37 sec) arms as the control group which indicates that the photic-CORT group had a normal level of anxiety. Interestingly, the fluoxetine-CORT group (n = 11) spent a similar amount of time in the open (9.94 ± 2.73 sec) and closed (270.32 ± 3.13 sec) arms as the only-CORT group. Although there was no statistical difference between the fluoxetine-CORT and control groups in terms of the amount of time spent in the open arms, statistical analysis revealed a significant difference between these two groups in the amount of time spent in the closed arms (p < 0.01, Scheffe’s *post hoc* test). In addition, the fluoxetine-CORT group spent less time in the center area than the other groups (F_3, 64_ = 4.94, p < 0.01, One-way ANOVA), and there was a significant difference in the amount of time in the center area between the fluoxetine-CORT (19.74 ± 1.86 sec) and control groups (38.50 ± 3.82 sec) (p < 0.01, Scheffe’s *post hoc* test). Taken together, these results demonstrate that rhythmic photic stimulation at alpha frequencies was more effective than fluoxetine treatment in terms of alleviating increased the anxiety levels of the only-CORT group.

### Mice receiving photic stimulation displayed normal locomotor activity in the open-field task

**Fig 3:**
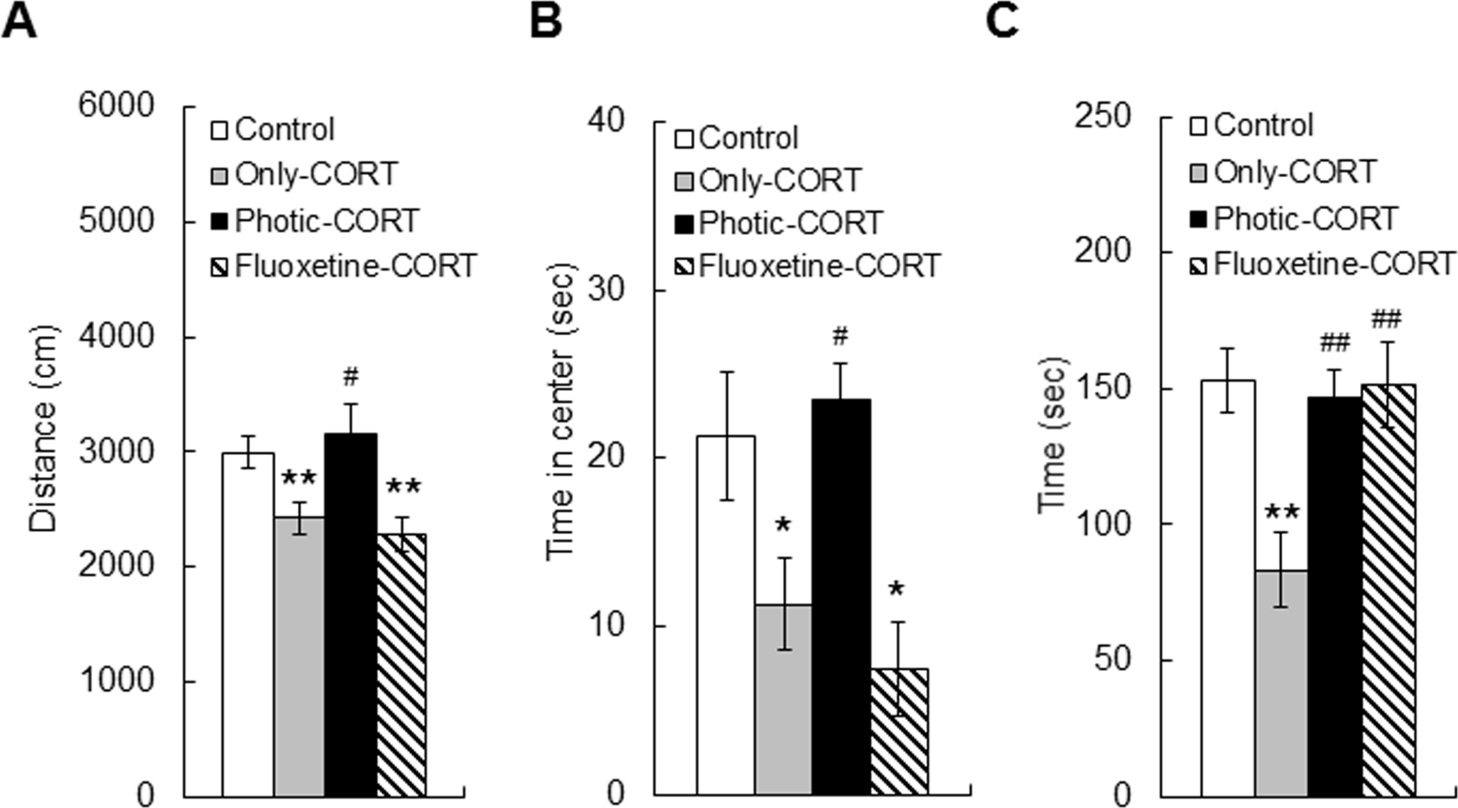
Effects of 10–12 days of treatment with photic stimulation or fluoxetine in the open-field and social interaction tasks in the CORT-induced mouse model of depression. (A–B) Open-field task: (A) Total distance moved in the open-field box, and (B) time spent in the center area of the open-field box. The photic-CORT and control groups exhibited similar distances moved and amounts of time in the center, whereas the only-CORT and fluoxetine-CORT groups showed a reduction in total distance moved and time spent in the center area compared with the control group. (C) Social interaction task: the photic-CORT and fluoxetine-CORT groups displayed similar interaction times compared with the control group, but the only-CORT group showed a reduced amount of interaction time compared with the other groups. *comparison with control, *p < 0.05, **p < 0.01; ^#^comparison with only-CORT group, ^#^*p* < 0.05, ^##^*p* < 0.01, Scheffe’s *post hoc* test.

Next, locomotor activity in the open-field box was assessed 10–12 days after the photic stimulation or fluoxetine treatment. A one-way ANOVA revealed significant differences in the distance moved (F_3, 64_ = 4.79, p < 0.01; Fig 3A) and the amount of time spent in the center area (F_3, 64_ = 5.76, p < 0.01; Fig 3B) among the control, only-CORT, photic-CORT, and fluoxetine-CORT groups. The only-CORT group (n = 20) (2427.43 ± 135.90 cm) moved a shorter distance than the control group (n = 19) (2993.71 ± 143.04 cm) (p < 0.01, Scheffe’s *post hoc* test, Fig 3A), whereas the photic-CORT group (n = 18) moved a similar distance (3153.43 ± 267.44 cm) as the control group. These findings indicate that there was normal locomotor activity in the photic-CORT group. Interestingly, the fluoxetine-CORT group (n = 11) (2279.32 ± 145.33 cm) moved a similar distance as the only-CORT group and that there was a significant difference in the total distance moved by the control and fluoxetine-CORT groups (p < 0.01, Scheffe’s *post hoc* test, Fig 3A).

The amount of time spent in the center area of the open-field box is a good indicator of anxiety levels. the photic-CORT (23.47 ± 2.19 sec) and control (21.31 ± 3.82 sec) groups spent similar amounts of time in the center area, whereas the only-CORT (11.31 ± 2.75 sec) and fluoxetine-CORT (7.48 ± 2.75 sec) groups spent less time in the center area compared with the control group (p < 0.05, Scheffe’s *post hoc* test, Fig 3B). This is consistent with the findings from the elevated plus maze task. Taken together, these results demonstrate that rhythmic photic stimulation at alpha frequencies, but not fluoxetine treatment, for 10–12 days can reverse the abnormal locomotor activity of CORT-exposed mice.

### Mice receiving photic stimulation or fluoxetine treatment exhibited normal social interactions

Social behavior was assessed with a social interaction task in which the mice were presented with an unfamiliar juvenile mouse after 10–12 days of photic stimulation or fluoxetine treatment. There were significant differences in social interaction time among the groups (F_3, 52_ = 7.72, p < 0.01, One-way ANOVA, Fig 3C). The only-CORT group (n = 18) (83.22 ± 13.77 sec) exhibited a significant reduction in social interaction time compared with the control group (n = 12) (153.31 ± 11.82 sec) (p < 0.01, Scheffe’s *post hoc* test, Fig 3C), but both the photic-CORT (n = 18) (146.88 ± 10.24 sec) and fluoxetine-CORT (n = 8) (151.58 ± 15.84 sec) groups displayed similar interaction times as the control group. This indicates that both rhythmic photic stimulation and fluoxetine treatment for 10–12 days reversed the impaired social behavior that was present in the only-CORT group.

### Photic stimulation enhanced the effects of fluoxetine in the FST

**Fig 4:**
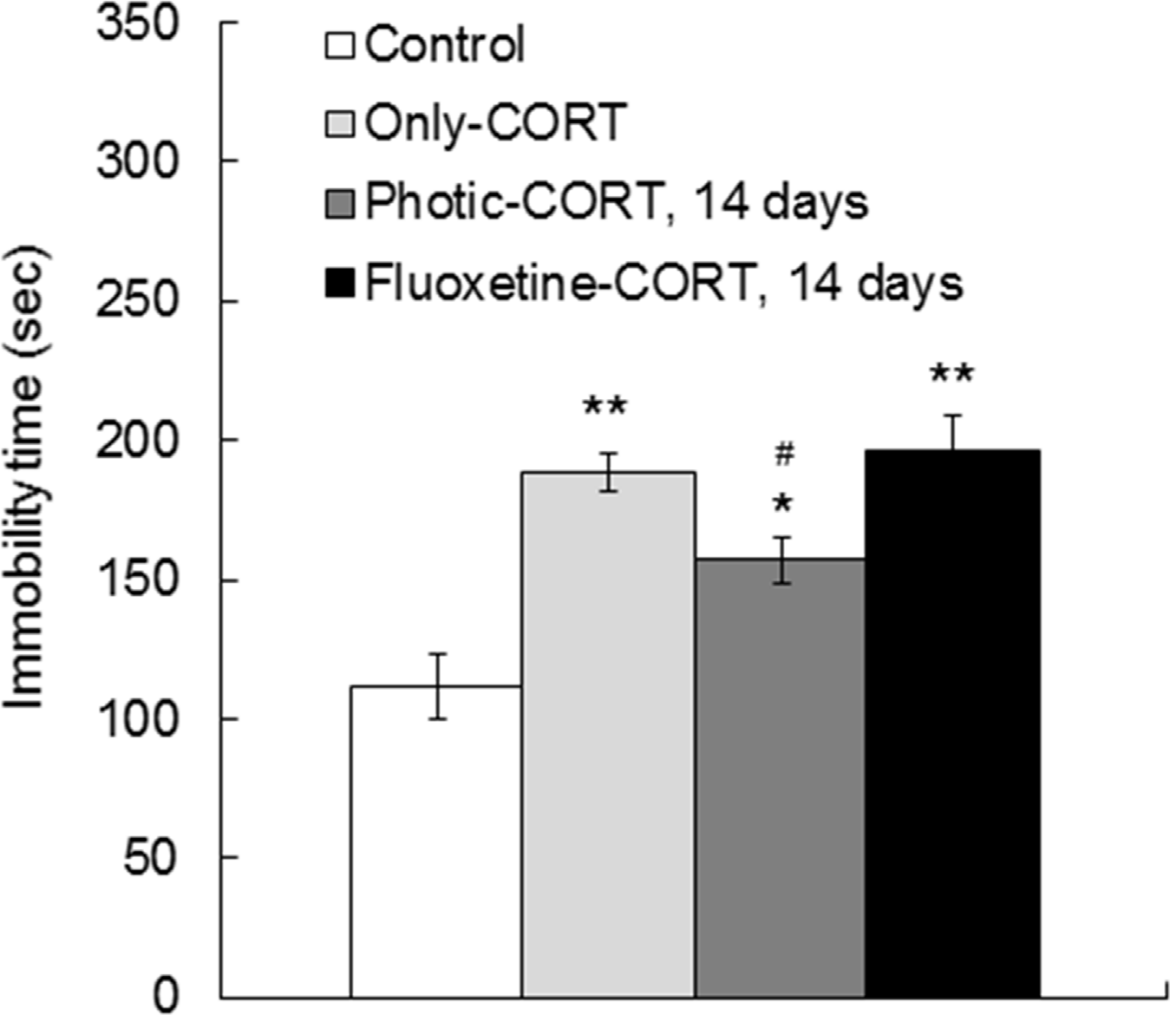
Effects of 8 or 14 days of treatment with photic stimulation and fluoxetine on the forced swim test in the CORT-induced mouse model of depression. The only-CORT group exhibited more immobility than the control group. Photic stimulation for 14 days but not 8 days resulted in a reduction in the immobility time compared with that of the only-CORT group. The fluoxetine-CORT group that received 14 days of treatment displayed a similar amount of immobility time compared with the only-CORT group. Notably, the co-administration of photic stimulation and fluoxetine for 8 days led to a reduction in immobility time compared with that shown by the only-CORT group. *comparison with control, **p < 0.01; ^#^comparison with only-CORT group, ^#^*p* < 0.05, Scheffe’s *post hoc* test.

The FST was used to assess behavioral despair, an indicator of depression-like behavior in mice, after 14 days of photic stimulation or fluoxetine treatment. There was a significant difference among the groups (F_3, 59_ = 15.02, p < 0.01, One-way ANOVA, Fig 4). The only-CORT group (n = 20) (188.14 ± 4.96 sec) exhibited increased immobility compared with the control group (n = 10) (112.07 ± 7.14 sec) (p < 0.01, Scheffe’s *post hoc* test, Fig 4), which suggests that CORT induced a higher level of despair. The photic-CORT (n = 22) (157.13 ± 8.53 sec) and fluoxetine-CORT groups (n = 11) (196.48 ± 12.96 sec) also had a longer immobility time than the control group (p < 0.05, Scheffe’s *post hoc* test). However, the immobility time of the photic-CORT group was shorter than those of the only-CORT and fluoxetine-CORT groups (p < 0.05, Scheffe’s *post hoc* test, Fig 4). The fluoxetine-CORT group displayed a similar immobility time as the only-CORT group. This result suggests that rhythmic photic stimulation at alpha frequencies alleviates despair-like behavior in an animal model of depression.

## Discussion

The present study investigated the effects of rhythmic photic stimulation on depression-like behaviors in a CORT-induced mouse model of depression using flickering and flashing lights at alpha frequencies. Additionally, the effects of flickering photic stimulation and fluoxetine, an SSRI antidepressant drug, in terms of the expression of depressive behaviors were compared. Photic stimulation improved the performance of mice that exhibited CORT-induced depressive behaviors in tests assessing anxiety, locomotor activity, social behavior, and despair-like behavior. Furthermore, photic stimulation over a short period (8 or 14 days) had a greater antidepressant effect on CORT-induced depression-like behaviors than fluoxetine.

Light therapy efficaciously treats the neuropsychiatric symptoms of various brain disorders and diseases including depression, schizophrenia, and Alzheimer’s disease or dementia [51–57]. However, although constant bright-light therapy has been well-studied, only a few studies have investigated the use of frequency-based flickering phototherapy for the treatment of depression. Among the brain oscillations that can be measured with an electroencephalography (EEG), the alpha rhythm is known to be associated with calmness, relaxation, and a peaceful yet alert and lucid mental state [58–61]; conversely, abnormalities in alpha oscillations often appear in patients with depression [62,63]. Rhythmic photic stimulation has been shown to alter brain oscillations, and this type of stimulation at alpha frequencies can evoke alpha oscillations in the brain [27,64–70]. Moreover, photic stimulation at alpha frequencies exerts beneficial effects on cognition and behavior [26–32]. Therefore, the alpha rhythm was used as the photic stimulation frequency in the present study. It is important to note that the CORT-induced mouse model of depression is associated with abnormal alpha activity in the frontal cortices of subjects (S1 Text and S1 Fig), but it is unclear whether the photic stimulation used in the present study induced or modulated the alpha oscillations. Further studies using EEG recordings to assess the entrainment of alpha oscillations with photic stimulation at alpha frequencies are needed to clarify this issue.

Subchronic treatment with fluoxetine for 1 or 2 weeks does not produce a substantial ameliorative effect on depression-like behaviors [35,39,40]. In the present study, the photic stimulation at alpha frequencies that was administered over a short period (8–14 days) produced antidepressant effects. Although photic stimulation alleviated depressive behaviors in the present CORT-induced mouse model of depression, these treatment effects may not persist. The photic-CORT and only-CORT groups exhibited similar behavioral performances in the elevated plus maze only 2 weeks after the cessation of the 8-day treatment period with photic stimulation, indicating remission in behavior (S2 Fig). In addition, the photic stimulation per se did not affect behavior of the control mice (S3 Fig). These results imply that the effects of photic stimulation are reversible and not pathological. However, the reversal of locomotor deficit by photic stimulation might underlie the antidepressant-like effect in the FST. Thus, it is thought that non-locomotor based tests for despair-like behavior are needed. In addition to the tests of depression-like behavior, the Y-maze task, which is a behavioral learning and memory task, was also conducted. Photic stimulation for 12–18 days did not improve CORT-induced behavioral dysfunction in the Y-maze (S1 Text and S4 Fig).

Depression is unlikely to result from the aberrant functioning of a single gene or individual brain region [71]. In fact, many studies have reported that numerous regions of the brain are affected by depression and that symptoms of depression are associated with dysregulation of distributed neural networks that encompass cortical regions rather than with the functional breakdown of a single discrete brain region [71–76]. As in humans, the CORT-induced mouse model of depression is also associated with abnormal neural networks [38]. The activation of the visual cortex by light or visual inputs can influence fronto-limbic structures including the prefrontal cortex, anterior cingulate cortex, basal ganglia, hippocampus, amygdala, and hypothalamus; not surprisingly, these regions are implicated in several affective disorders, including depression [12,74,77–79]. Flickering photic stimulation synchronizes brain activity, and it is thought that the synchronization of brain oscillations can result in temporal integration, the binding of salient stimulus features across different sensory cortices, increased spatial discreteness, and somatotopical specificity [27,64–70]. Additionally, the synchronization of brain oscillations can increase the flow of information among brain regions, facilitate neuronal communication, and play a crucial role in cortical integration and perception/cognition [59,80–85]. Thus, chronic rhythmic photic stimulation may help restore the functioning of abnormal neural networks in subjects with depression. Moreover, a number of studies employing neuroimaging, electrophysiological, and biochemical measurement tools have demonstrated that light stimulation induces positive changes in cerebral blood flow and the brain metabolism of neurotransmitters or neuromodulators, including melatonin, serotonin, and cortisol, which are also impaired in depression [13,86–96].

In conclusion, the present study demonstrated that rhythmic photic stimulation at alpha frequencies produces antidepressant effects in a CORT-induced mouse model of depression. More specifically, rhythmic photic stimulation at alpha frequencies rapidly improved behavioral dysfunction compared with fluoxetine. It has been suggested that frequency-based rhythmic stimuli consisting of light, sound, or both can influence brain activity and produce positive behavioral outcomes [27]. The present findings support the efficacy of adjuvant light therapy when used in conjunction with antidepressant drugs.

## Supporting Information

S1 Fig Altered alpha rhythms in the CORT-induced mouse model of depression. The histogram represents normalized EEG power; the EEG power values of subjects exposed to the CORT-induced mouse model of depression (n = 9) decreased in the alpha-band frequencies compared with those of the control group (n = 10). *p < 0.05, Student’s *t*-test.
(TIF)

S2 Fig Remission in behavior on the elevated plus maze task in the CORT-induced mouse model of depression. The elevated plus maze task was performed 2 weeks after the cessation of 8 days of treatment with photic stimulation. The photic-CORT group (n = 10) spent similar amounts of time in the open and closed arms compared with the only-CORT group (n = 19). **p < 0.01, Scheffe’s *post hoc* test.
(TIF)

S3 Fig The control group that received photic stimulation (photic-control group) normally behaved. (A) Elevated plus maze task: the photic-control group (n = 10) spent a similar amount of time in the open and closed arms as the nonphotic-control group (n = 15). (B–C) Open-field task: (B) Total distance moved in the open-field box, and (C) time spent in the center area of the open-field box. Two control groups (photic, n = 10; nonphotic, n = 19) exhibited similar distances moved and amounts of time in the center. (D) Social interaction task: the photic-control group (n = 9) displayed similar interaction times compared with the nonphotic-control group (n = 12). (E) FST: two control groups (photic, n = 10; nonphotic, n = 10) displayed a similar immobility time.
(TIF)

S4 Fig Effects of photic stimulation on the Y-maze task in the CORT-induced mouse model of depression. The only-CORT group (n = 12) showed impaired performance by spending the same amount of time in novel and familiar arms, whereas the control group (n = 10) spent more time in the novel arm (**p < 0.01, one-way ANOVA). Additionally, the photic-CORT group (n = 12) that received photic stimulation for 12 or 18 days showed no preference for the novel arm, indicating that photic stimulation did not affect learning and memory performance in the Y-maze. 1: start, 2: known; 3: novel arm.
(TIF)

S1 Text Supporting text. (DOCX)
