# Supplementary figures and images for: Rhythmical Photic Stimulation at Alpha Frequencies Produces Antidepressant-Like Effects in a Mouse Model of Depression
Source: PLoS One. 2016 Jan 4;11(1):e0145374. doi: 10.1371/journal.pone.0145374 (PMC4699699; doi:10.1371/journal.pone.0145374)

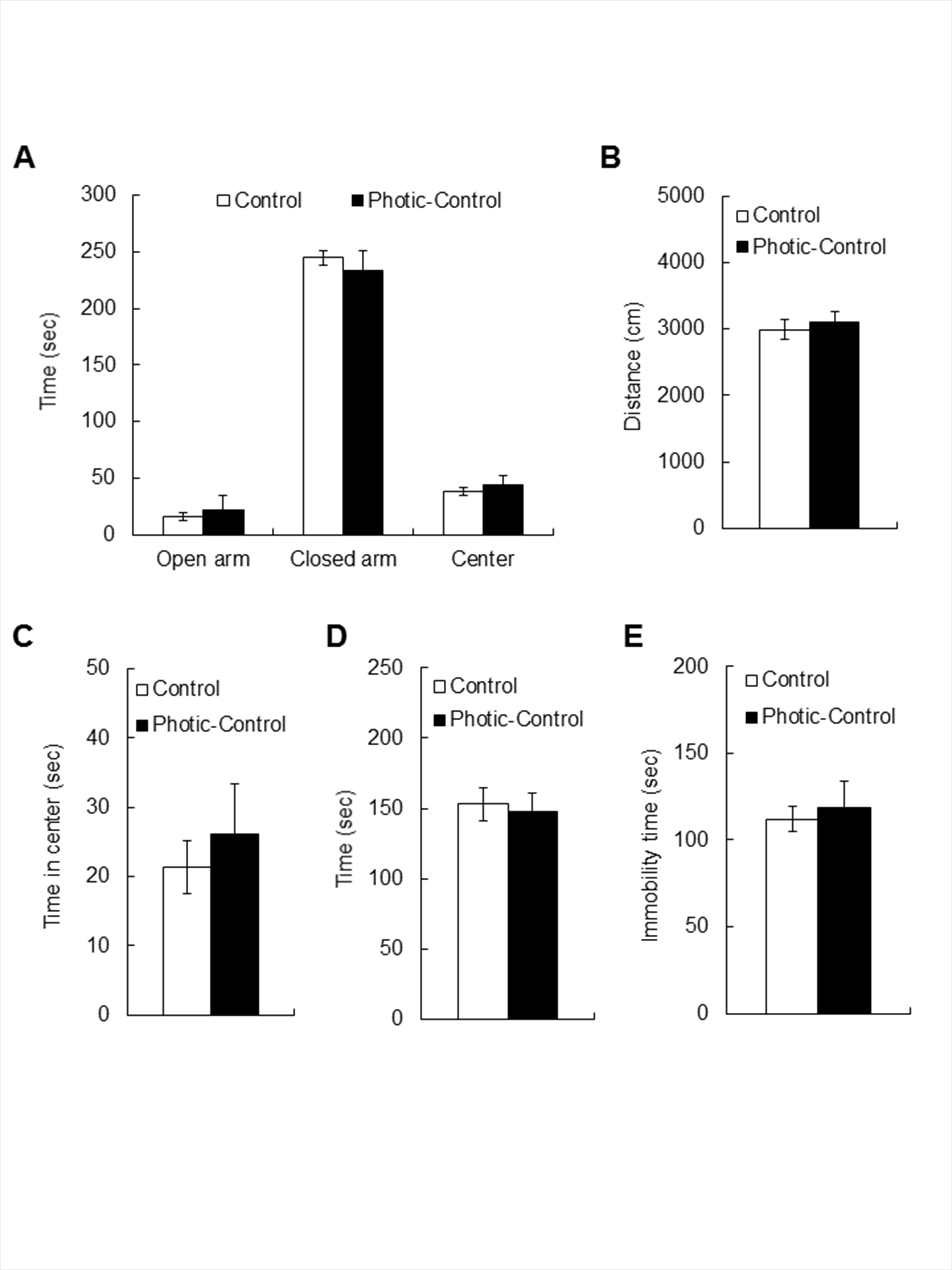

Supplement: S3 Fig — (A) Elevated plus maze task: the photic-control group (n = 10) spent a similar amount of time in the open and closed arms as the nonphotic-control group (n = 15). (B–C) Open-field task: (B) Total distance moved in the open-field box, and (C) time spent in the center area of the open-field box. Two control groups (photic, n = 10; nonphotic, n = 19) exhibited similar distances moved and amounts of time in the center. (D) Social interaction task: the photic-control group (n = 9) displayed similar interaction times compared with the nonphotic-control group (n = 12). (E) FST: two control groups (photic, n = 10; nonphotic, n = 10) displayed a similar immobility time. (TIF) [file pone.0145374.s003.tif]
