# Supplementary material for: Rhythmical Photic Stimulation at Alpha Frequencies Produces Antidepressant-Like Effects in a Mouse Model of Depression
Source: PLoS One. 2016 Jan 4;11(1):e0145374. doi: 10.1371/journal.pone.0145374 (PMC4699699; doi:10.1371/journal.pone.0145374)
Supplement: S1 Text — (DOCX) [file pone.0145374.s005.docx]

**S1 Text**

**Supporting Text**

**In vivo electrophysiology for EEG**

EEG surgery and recording were conducted as described previously [[38](#_ENREF_38),[44](#_ENREF_44),[47](#_ENREF_47)]. Animals underwent EEG surgery immediately after the CORT treatment. Animals were anesthetized by intraperitoneal injection of ketamine (90 mg/kg) and xylazine hydrochloride (40 mg/kg). Electrode implantation was performed with a stereotaxic apparatus (Kopf Instruments, Tujunga, CA, USA). EEG recordings were obtained with a tungsten electrode (0.005 in, 2 MΩ), positioned in frontal cortex (AP +1.5 mm, L +0.2 mm, and DV –1.0 to –1.1 mm), based on a mouse brain atlas. A reference electrode was inserted on the skull above the cerebellum. The electrodes were fixed to the skull with cyanoacrylate adhesive and dental acrylic cement. After 7-day recovery, EEG recordings were combined with video monitoring, and EEG-video recording data were obtained continuously for a day. EEG activity was recorded after the signal was amplified 1200-fold, band pass-filtered at 0.1-70 Hz, and digitized at a sampling rate of 400 Hz using a digital EEG system (Comet XL, Astro-Med, West Warwick, RI, USA). Continuous EEG signals from the animals for 1 min, in which they were in a resting state (i.e., awake and no movement), were analyzed. The relative normalized power at each individual frequency was presented as a fraction of the sum of powers at all frequencies of delta (1.5–4 Hz), theta (4.5–8 Hz), alpha (8.5–12 Hz), beta (12.5–30 Hz), and gamma (30–60 Hz) [[38](#_ENREF_38),[47](#_ENREF_47)]. The electrophysiological data were analyzed offline using PSG Twin 4.2 (Astro-Med) and pClampfit 10.2 (Axon Instruments, Union City, CA, USA).

**Y-maze task**

Y-maze test was performed as described previously [[97](#_ENREF_97)]. The Y-maze apparatus consisted of three identical arms. Each arm was 25 cm long, 5 cm wide, and 14 cm high. Briefly, one of the arms of the maze was closed and mice were placed randomly into one of the other arms (start arm) and allowed to explore the maze for 10 min (training session). An hour later, mice were replaced in a start arm, and then allowed to freely explore all three arms for 5 min (test session). Retention times (duration) in each arm were used to assessed spatial memory. Results are presented as ratios of the amount of time spent in each arm over the total time spent in the three arms.
